# Understanding Actions of Others: The Electrodynamics of the Left and Right Hemispheres. A High-Density EEG Neuroimaging Study

**DOI:** 10.1371/journal.pone.0012160

**Published:** 2010-08-13

**Authors:** Stephanie Ortigue, Corrado Sinigaglia, Giacomo Rizzolatti, Scott T. Grafton

**Affiliations:** 1 4D Brain Electrodynamics Laboratory, Department of Psychology, UCSB Brain Imaging Center, Institute for Collaborative Biotechnologies, University of California Santa Barbara, Santa Barbara, California, United States of America; 2 Laboratory for Advanced Translational Neuroscience, Department of Psychology, Central New York Medical Center, Syracuse University, Syracuse, New York, United States of America; 3 Department of Philosophy, University of Milan, Milan, Italy; 4 Department of Neuroscience, University of Parma, Parma, Italy; 5 Istituto Italiano di Tecnologia, Unità di Parma, Parma, Italy; Georgetown University, United States of America

## Abstract

**Background:**

When we observe an individual performing a motor act (e.g. grasping a cup) we get two types of information on the basis of how the motor act is done and the context: what the agent is doing (i.e. grasping) and the intention underlying it (i.e. grasping for drinking). Here we examined the temporal dynamics of the brain activations that follow the observation of a motor act and underlie the observer's capacity to understand what the agent is doing and why.

**Methodology/Principal Findings:**

Volunteers were presented with two-frame video-clips. The first frame (T0) showed an object with or without context; the second frame (T1) showed a hand interacting with the object. The volunteers were instructed to understand the intention of the observed actions while their brain activity was recorded with a high-density 128-channel EEG system. Visual event-related potentials (VEPs) were recorded time-locked with the frame showing the hand-object interaction (T1). The data were analyzed by using electrical neuroimaging, which combines a cluster analysis performed on the group-averaged VEPs with the localization of the cortical sources that give rise to different spatio-temporal states of the global electrical field. Electrical neuroimaging results revealed four major steps: 1) bilateral posterior cortical activations; 2) a strong activation of the left posterior temporal and inferior parietal cortices with almost a complete disappearance of activations in the right hemisphere; 3) a significant increase of the activations of the right temporo-parietal region with simultaneously co-active left hemispheric sources, and 4) a significant global decrease of cortical activity accompanied by the appearance of activation of the orbito-frontal cortex.

**Conclusions/Significance:**

We conclude that the early striking left hemisphere involvement is due to the activation of a lateralized action-observation/action execution network. The activation of this lateralized network mediates the understanding of the goal of object-directed motor acts (mirror mechanism). The successive right hemisphere activation indicates that this hemisphere plays an important role in understanding the intention of others.

## Introduction

Although humans interact mostly verbally, non-verbal interactions are also fundamental in social life. We continuously observe our conspecifics and from their facial expression, body posture, and the way in which they act upon objects we are able to understand their emotions, mood and intentions [1], [2], [3], [4].

What are the mechanisms that allow us to become aware of the mental states of others? There is a long tradition claiming that humans understand what others are doing by means of their capability to attribute a causal role to others' internal mental states [5], [6].

In recent years, however, neurophysiological evidence has shown that the actions of others can be understood without exploiting these meta-representational abilities. Single neuron recordings in the monkey and brain imaging and electrophysiological non-invasive techniques (Transcranial Magnetic Stimulation, TMS; Electroencephalogram, EEG; Magneto-Encephalogram, MEG) in humans showed that primates are endowed with a mechanism -the mirror mechanism- that matches the observed motor acts done by others on the observer's motor representations of the same motor acts [7], [8], [9], [10]. Because the observer knows his/her own actions and the way to achieve them, the occurrence of a neural pattern similar to that the observer generates during voluntarily motor acts enables him/her to recognize the motor act he/she is observing.

These data do not deny, of course, that meta-representational abilities might play a role in action understanding or in reasoning about the observed motor action [11], [12], [13], [14], [15], [16]. They indicate, however, that mentalizing is neither the *sole*, nor the *primary* way of understanding others' actions.

While the early studies on mirror neurons in monkeys [17], [18] described a mechanism allowing the observer to understand *what* an individual was doing in a given moment (e.g. grasping, ripping, holding), more recent data indicate that the mirror mechanism might also account for the capacity to understand *why* the agent was doing it, that is the agent's *motor intention*. Fogassi et al. (2005) showed that, in the inferior parietal lobule (IPL) of the monkey, the majority of motor neurons discharge during a motor act (e.g., grasping) only if this act is embedded in a specific chain of motor acts (e.g., grasping-for-eating). Most interestingly, many of these “action-constrained” motor neurons have mirror properties firing in relation to the observation of a motor act provided that this act is part of a specific action [19]. By virtue of these “action-constrained” mirror neurons and their motor connections, the observation of the initial motor act of an action evokes in the observer's brain an internal motor copy of the whole action that the agent is going to perform, and this enables her/him to understand the intention underlying the observed motor act.

Brain imaging data indicate that the mirror mechanism also plays a role in intention understanding in humans [20], [21]. Furthermore, by using EMG, it has been shown that the observation of the initial motor act (e.g. grasping) of an intentional action (grasping a piece of food for eating it) evokes in human observers a motor copy of the action the agent is going to perform. This motor copy allows the observer to grasp the agent's intention, without bringing into plays specialized inferential processes of mentalistic rationalization [22].

The existence of two separate, although interconnected, mechanisms underlying the what- and why- of understanding was confirmed by a behavioral study in humans. Typically developing children (TD) and children with autism were asked to recognize an everyday life motor act and, in a subsequent condition, to tell the experimenter why the observed motor act was performed [23]. The data showed that while TD children had no problems with both tasks, children with autism recognized *what* another individual was doing [21], [24], [25], [26], [27], but frequently failed in understanding *why* that individual was doing it.

This dissociation between the capacity to understand the what and why of a motor act suggests that, even when action and intention understanding does not require specialized inferential reasoning on others' mental states, different, although interconnected neural mechanisms, are involved in the brain of the observer. Nothing, however, is known on the *temporal-spatial* events leading from the observation of a simple motor act, like grasping a mug, to the understanding of the intention underlying it.

The aim of the present study was to describe the temporal sequence of brain activations that allow the observation of a motor act done by another individual and underlie the observer's capacity to understand what the agent is doing and why. To this purpose we presented scenes where the goal and intention of the observed motor acts could be understood on the basis of the relation between a hand and an object or by the context in which the motor acts were performed. Furthermore, in some cases the purpose of the hand motor act was transparent, while in others it was opaque and could be only guessed.

As a technique, we used high-density electrical neuroimaging, which combined a cluster-analysis of brain topography (a technique elaborated by Lehmann, [28]) with inverse solutions [29], [30], [31], [32]. This technique has the advantage of providing high temporal and a relatively high spatial resolution about stimulus information processing by unraveling the temporal sequence of the brain topographies (brain microstates) that are stable after a stimulus onset and the spatial location of the brain generators of these brain topographies [31], [32], [33], [34], [35]. This technique has proven to be a reliable research tool during the past decade [35], [36], [37], [38], [39], [40].

## Materials and Methods

### Participants

The experiments were carried out on 20 volunteers (fifteen men, five women; mean age of 24 years old; age range of 18–44 years old). All were right-handed, as ascertained by the Edinburgh Handedness Inventory [41]. They had normal or corrected-to-normal visual acuity. None had prior or current neurological or psychiatric impairment. Prior to participation, all participants provided a written informed consent to take part in the study that has been approved by the University of California Santa Barbara's Institutional Review Board.

### Stimulus sequence

**Figure 1:**
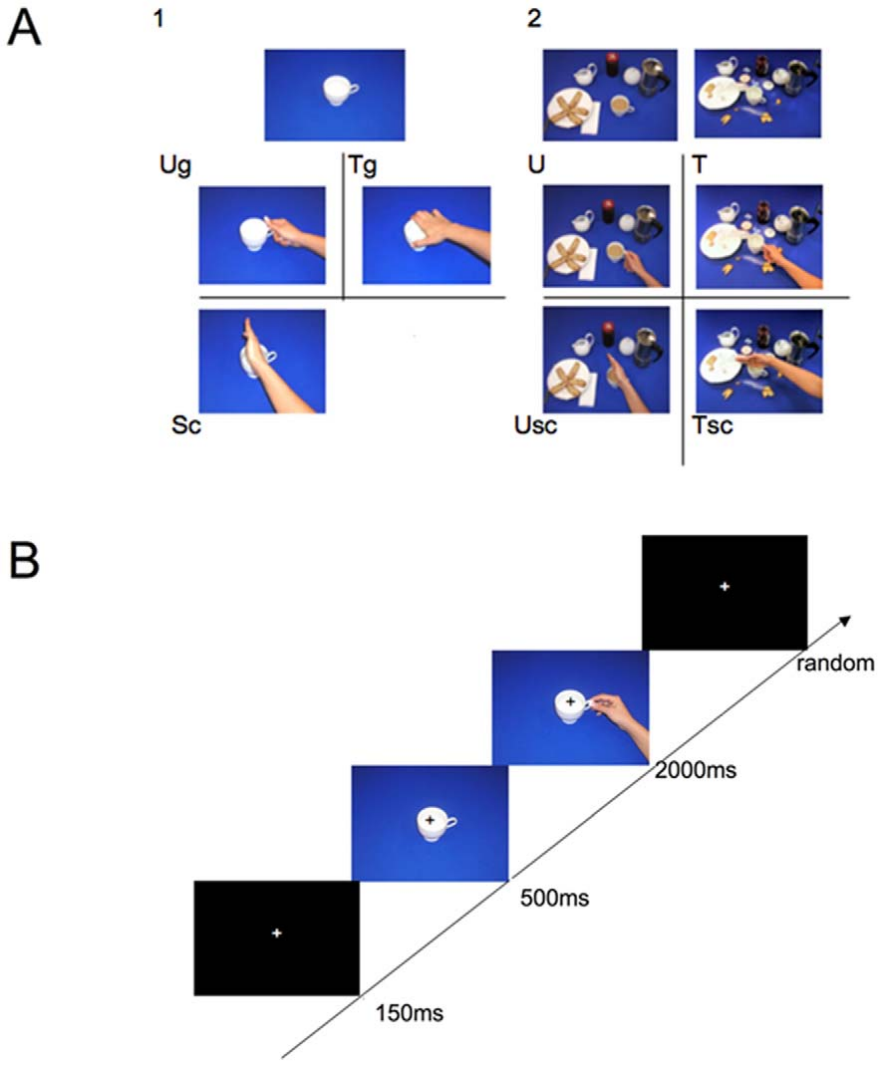
Stimuli and experimental design. **A**. Exemplars of the used stimuli. 1: No context condition. Participants observed two pictures in sequence. The first showed an object (e.g., a cup) without any context, the second a hand interacting with that object. Three types of hand-object interactions were presented: a hand grasping the object as for using it (Ug); a hand grasping an object as for moving it (Tg); a hand touching an object without any obvious purpose (Sc). 2: “Context” condition. As in the previous condition, participants saw two pictures in sequence. The first showed an object embedded in a context (upper row). The second one showed a hand grasping that object. The context allowed the observer to decide whether the agent's intention was to use the object or to move it (U and T, middle row). A simple contact of the hand with the object was also presented in both the contexts (Usc: use context, simple contact; Tsc; transport context, simple contact; upper right, lower row). **B**. Procedure. Each trial consisted of the following sequence: fixation cross; object alone or within a context hand-object interaction; fixation cross. The sequence shown in figure illustrates the use grip (Ug) in No context condition.

Participants viewed a series of two-frame video-clips (Figure 1A). Every video clip consisted of the following sequence: a fixation cross (150 ms), an object without context or an object within two different contexts (T0; 500 ms), and a hand-on-object action with or without contexts (T1; 2000 ms; Figure 1B). T1 (hand-object interaction) showed a hand grasping or touching the object. Because of this sequence, all participants identified the hand-object interaction as the initial part of an action (see Debriefing session). The gap between the first (T0) and the second (T1) stimulus was very short (one refreshed screen, see [35], [42]). In this way the continuous images sequence determined the perception of an action. The presentation of the each couple of stimuli (T0 and T1) was interspersed with the presentation of a black screen with a fixation cross in its center (Figure 1B). Inter-trial intervals (ITI) ranged from 1000 to 2000 ms with steps of 100 ms varying randomly in different trials (ITI = 1000, 1100, 1200, …. 2000).

### Paradigm

**Figure 2:**
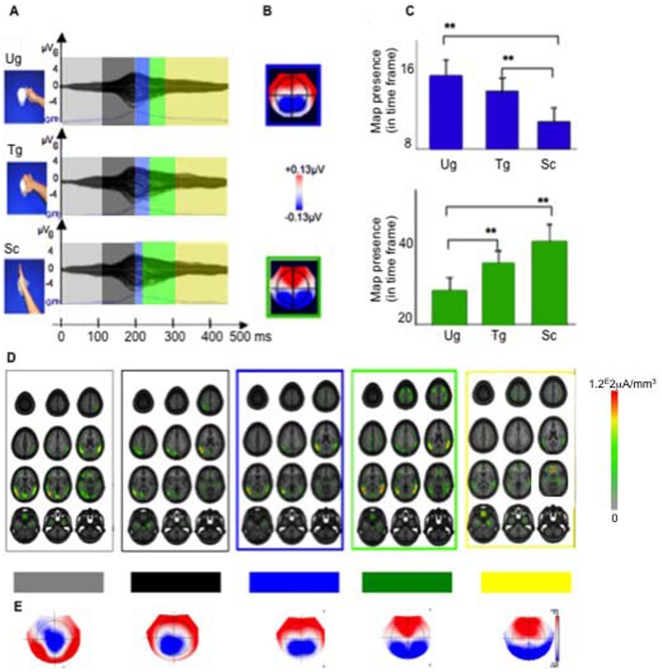
No Context condition. Visual event-related potentials (VEPs) and brain microstates. **A**. Group-averaged VEPs elicited by the presentation of use grip (Ug), transport grip (Tg) and simple contact (Sc) stimuli. Stimulus exemplars of the three classes of stimuli are shown on the left side of the panel. The topographic cluster analysis identified five distinct microstates (colored bars) in the 500 ms following stimulus presentation. Two microstates (blue and green bars, respectively), although present following presentation of all three types of stimuli, had different duration depending upon the stimulus type. **B**. Segmentation maps of the two microstates (Microstate blue frame, Microstate green frame) that showed different duration according to the presented stimulus type. The maps are plotted with the nasion upward and right ear on the right side (scale indicated). Blue areas depict negative potentials and red areas depict positive potentials. **C**. The statistical significance of Microstate 3 and 4 was tested by means of a fitting procedure based on the spatial correlation between microstates obtained from the group-averaged VEPs and the single-subject VEP data. Blue columns: Microstate 3. Note the more prolonged activity for use and transport grip (Ug and Tg,) than for simple contact actions (Sc). Green columns: Microstate 4. Note the shorter activity in response to use grip (Ug) than in response to the other two stimuli. Error bars indicate standard deviation. Asterisks indicate significant differences (**, *P*<0.01) between conditions for a given microstate. **D**. Localization of the intracranial brain generators as estimated with LORETA inverse solution. Twelve transaxial brain sections are presented. Their Talairach z values, from left to right and from top to bottom, are: 72, 64, 49, 42, 31, 22, 16, 7, −6, −10, −32, −38. Group-averaged source estimations were calculated over each time interval and all conditions. The localizations are framed with the same color code as the corresponding microstates in A. **E**. Segmentation maps of the all microstates. Conventions as in B.

Participants were tested in two main experimental conditions: “No Context” (Figure 1A, 1) and “Context” (Figure 1A, 2).

In the “No Context” condition three types of hand-object interaction (T1), differing for the intention underlying them, were used. These three types of hand-object interactions were the following: a) a right hand grasping an object for *purpose of use* (Use grip, Ug); b) a right hand grasping an object for *moving* it (Transport grip, Tg); c) a right hand *touching* an object. In this last case no obvious purpose of the observed motor act could be recognized (Simple contact, Sc). Exemplars of the three kinds of hand-object interactions are shown in Figure 1A1.

In the “Context” condition, the participants saw an establishing scene (T0 frame) suggestive of a certain type of behavior (Figure 1, A2), and subsequently a hand-object interaction (T1) embedded in that scene. There were two types of hand-object interactions. The first showed a hand grasping an object with the grip being the one typically employed for the use of that object. However, the context in which the grip was performed indicated whether the use interpretation was the correct one (Figure 1, A2, U) or whether the most likely intention of the agent was that of moving the object towards another position (Figure 1, A2, T). The second type of hand-object interaction consisted of a hand touching an object in two different contexts (Figure 1, A2; use context, simple contact, Usc; transport context, simple contact, Tsc). In neither context, the purpose of touching could be derived from the hand-object interaction.

### Participants' instruction

During the EEG recordings, the participants received explicit instructions to observe carefully the video-clips, and to try to understand the intention behind the observed hand-object interactions (T1) for both the “Context” and “No Context” conditions. In order to avoid eye movements, participants were asked to fixate the central visual cross during the whole experiment. Before EEG recordings, every participant was familiarized with all actions for 3 minutes. Participants were informed that some of the hand-object interactions could have the following intentional meaning: to transport the grasped object or to use the grasped object. Participants were also informed that some of the hand-object interactions could be devoid of an immediate intentional meaning. During EEG recordings, participants were required to not give any overt responses. No reaction times were recorded during the EEG session to avoid any motor interference with EEG activity.

### Debriefing session

At the end of the experimental session all participants were debriefed. During this debriefing session, behavioral responses were collected by asking participants to write down their responses on a piece of paper. This information was collected for every set of actions and every condition. As in the EEG session, the participants were instructed to try to understand the intention behind the observed hand-object interactions (T1) for both the “Context” and “No Context” conditions. Similarly, they were also informed that some of the hand-object interactions could have the intentional meaning of “transporting” the grasped object or of “using” it. Participants were also told that some of the hand-object interactions (e.g. touching) could be devoid of a clear intentional meaning (i.e., simple contacts: Sc, Tsc or Usc).

The behavioral results showed that all stimuli that enabled the observer to understand the agent's intentions were correctly rated. Specifically, in the “No Context” condition 99% of the responses of the participants correctly indicated the intention behind the use grip (Ug), and 98% percent of the responses of the participants correctly indicated the intention behind the transport grip (Tg). No difference was observed between Ug and Tg (*p* = 0.93). Also, 100% of the responses of the participants correctly indicated the hand-object interactions that were devoid of a clear intentional meaning (i.e., Sc) as such.

In the “Context” condition, 100% of the responses of the participants correctly indicated the stimuli for U, T, Usc, and Tsc.

A twelve-point Likert Scale ascertained participant's response certainty. In the “No Context” condition, the averaged certainty scores were 10.88 for Ug and 9.67 for Tg and. A ceiling effect (i.e., maximum score) was observed for Sc. In other words, simple contact stimuli were perfectly identified as being “simple touches with no obvious intentional meaning”. In the “Context” condition, the averaged certainty scores were 11 for the use of the object (U) and 10 for the transport of the object (T). No significant difference was observed between the two conditions. A ceiling effect (i.e., maximum score) was also observed in the “Context” condition for Usc and Tsc i.e., simple contact stimuli were perfectly identified as being “simple touches with no obvious intentional meaning”.

### Stimuli

In the “No Context” condition, 20 grasping actions (10 Ug and 10 Tg) and 10 touching actions (Sc) were presented intermixed among them. All actions were performed on the same 10 objects: screwdriver, banana, phone, glasses, pencil, coffee cup, scissors, hammer, pizza, and iron.

In the “Context” condition, 12 grasping actions (6 U and 6 T) and 12 simple contact actions (6 Usc and 6 Tsc) were presented, intermixed among them. The actions were performed on 6 objects that were also employed in the “No Context” condition. They were: coffee cup, scissors, pizza, phone, hammer, and iron. The reason for this selection was that grasping actions on the six chosen objects could be performed plausibly within two different ecological contextual backgrounds.

The grasping presented in the “Context” condition was of one type only, and namely the use grip (Ug) of the “No Context” condition. Ug was chosen because is the prototypical grip for a given object. Using the same grip in the “Context” and “No Context” condition allowed us to see how the context influenced the intention comprehension during the observation of the same motor act.

### Apparatus

Visual stimuli were presented on a PC computer using Cogent 2000 (http://www.vislab.ucl.ac.uk/Cogent2000/index.html) running in Matlab 7.0.1 under Windows XP. Participants were comfortably seated 150 cm away from a PC computer screen in which video clips were presented centrally. A total of three experimental blocks of “No Context” condition and five experimental blocks of “Context” condition were randomly presented throughout the whole experimental session. In each condition (“No Context”, and “Context”), grasping and simple contact actions were intermixed. A total of 240 trials were administered per condition (“No Context”, and “Context”), which took up to a total of 40 minutes including breaks between each block.

### Electrophysiological recordings

Electroencephalogram (EEG) was recorded from 128 AgCl carbon-fiber coated electrodes using an Electric Geodesic Sensor Net® (GSN300; Electrical Geodesic Inc., Oregon; http://www.egi.com/). EEG was digitized at 500 Hz, and band-width filtered at 0.01–200 Hz, with the vertex electrode (Cz) serving as an on-line recording reference.

### Data Analysis

#### First-level electrophysiological data analysis

Electrophysiological data were first analyzed at the individual level. Raw data of each participant were imported and analyzed in Cartool software (version 3.33; http://brainmapping.unige.ch/Cartool.htm). All trials were submitted to an automated threshold rejection criterion of 100 µV and visually inspected for oculomotor (saccades and blinks), muscles, and other artifacts. Channels with corrupted signals were interpolated using a three-dimensional spline procedure [43]. Surviving epochs of EEG (from −100 to 500 ms post-stimulus onset) were averaged for each experimental condition for each participant to calculate the visual event-related potentials (VEPs). Two VEPs were calculated for each participant: the first was time-locked to the onset of the first presented picture (T0, i.e., the picture showing objects with no hand-object interaction), the second with the onset the second presented picture (T1; i.e., the picture showing a hand-object interaction). For the data time-locked to the first picture (T0), three VEPs were computed for each participant, one for each condition: 1) object without context; 2) object within a context suggesting the object use; 3) object within context suggesting object transport (See Supporting Information, File S1, and Figure S1 for further details). For data time-locked to the second picture (T1), seven VEPs were computed for each participant, one for each of the three types of stimuli of the “No Context” conditions (Ug, Tg, and Sc) and one for each of the four types of stimuli in the “Context” condition (U, T, Usc, and Tsc).

These VEP data were baseline corrected from −100 to 0 and band-pass filtered between 1 and 30 Hz. Then, VEP data were recalculated off-line against the average reference, and normalized to their mean global field power (i.e., GFP [44], [45]). This GFP measure, first introduced by Lehman and Skrandies (1980), is equivalent to the spatial standard deviation of the scalp electric field calculated as the root mean square across the average-referenced electrode values at a given instant in time [34]. GFP yields larger values for stronger electric fields [32], [44].

All individual VEP data were then group-averaged using the following procedure. First, three group-averaged VEPs were computed across participants for data time-locked to the first picture (T0; one group-averaged VEP for object without context; one for object within a context suggesting object use; and one for object within context suggesting object transport). Then, seven group-averaged VEPs were computed across participants for data time-locked to the second picture (T1; one for each of the three types of stimuli of the “No Context” condition (Ug, Tg, and Sc) and one for each of the four types of stimuli in the “Context” condition; U, T, Usc, and Tsc). Because the main aim of the present experiment was to determine the temporal dynamics leading to the understanding of others' motor intentions (*why*), we focused on the grand-averaged VEPs synchronized to the onset of the second picture (T1) of the video clips, i.e., picture showing the hand-object interaction.

#### Second-level electrophysiological data analysis

Group-averaged VEP data were subsequently processed using a space-oriented brain electric field analysis [28]. This method is based on the notion of functional *brain microstates* introduced in the 1980's by Lehman [28]. It is based on the empirical observation that the electric brain activity does not vary randomly over time after a stimulus onset, but, rather, that some brain topographies remain stable over time (from tens to hundred milliseconds, [33]). Each stable brain topography (named brain microstate) is sustained by a specific brain network and reflects a specific functional brain state [28], [32], [33].

A way to identify the microstates is by carrying out a cluster analysis performed on the group-averaged VEPs [46]. This analysis was applied here on the group-averaged VEPs to determine the existence of stable microstates in the different experimental conditions. To identify the start and the end of each microstate, a standard pattern analysis was employed using the grand-mean VEPs of each condition [47]. This cluster analysis uses a hierarchical agglomerative cluster-algorithm to identify the predominant topographies (i.e., the microstates) and their sequence within a data set. A modified Krzanowski-Lai criterion determined the optimal number of microstates that explains the whole group-averaged data set (i.e., the minimal number that best accounts for the data set). Importantly, this cluster analysis is reference-free, and insensitive to amplitude modulation of the same scalp potential field across conditions, since normalized maps are compared. As in previous studies [34], [36], [37], [38], [47], this cluster analysis was performed across time and experimental conditions in order to determine whether and when different experimental conditions engaged distinct configurations of intracranial generators. Then, the appearance and sequence of the microstates identified in the group-averaged VEP data was statistically verified in the single-subject VEPs of the individual participants by means of a fitting procedure based on the spatial correlation between the template brain topographies obtained from the group-averaged level and the single-subject VEP data [48]. In other words, each microstate was compared with the moment-by-moment scalp topography of the individual participants' VEPs from each condition by strength-independent spatial correlation [34]. Thus, for each time point of the individual participant's VEPs, the scalp topography was compared to all microstates and was labeled according to the one with which it best correlated spatially [34], [48]. Because the pre-stimulus period (−100 to 0) was baseline corrected, we restricted our microstate analyses to the initial 500 ms post-stimulus period in terms of the spatio-temporal characteristics of the global electric field on the scalp (brain microstates). The optimal number of microstates was fitted into the original data for each subject, using a competitive fitting procedure [38], [49]. From this “fitting” procedure, we determined the total amount of time (i.e., duration) a given topography was observed for a given condition across participants. These latter values, which represent the frequency with which a given microstate was observed within a given time period for each experimental condition, were then subjected to a repeated measure ANOVA. It is important to note that this labeling procedure is not exclusive, such that a given period of the VEP for a given participant and stimulus condition is often labeled with multiple microstates. As such, the results of the labeling reveal if the VEP from a given experimental condition is more often described by one microstate vs. another, and therefore if different generator configurations better account for particular experimental conditions. Results were accepted as significant at *p*<0.05.

As a final step, we estimated the sources in the brain that gave rise to each of the microstates, using a distributed linear inverse solution. The inverse matrices applied here were based on a low-resolution brain electromagnetic tomography (LORETA) model of the unknown current density in the brain [29], [50]. Since LORETA belongs to the class of distributed inverse solutions, it is capable of dealing with multiple simultaneously active sources of a priori unknown location. LORETA method has been quoted in several publications and has been validated theoretically and empirically [30], [46], [50]. The applied version of LORETA was used with a lead field (solution space) calculated on a realistic head model using SMAC [51] on an average brain model provided by the Montreal Neurological Institute (MNI). Our head model included 3005 solution points, selected from a 6×6×6 mm grid equally distributed within the gray matter. Source estimations were rendered on the MNI/McGill average standard brain supplied by Cartool. As an output, this approach provides current density measures (in µA/mm^3^) at each solution point. The results of the abovementioned topographic pattern analysis defined the time period (i.e., microstate) when intracranial sources were estimated with the distributed source inverse solution (LORETA). Although LORETA provides one current source density maximum for each microstate, it may also, as a distributed inverse solution, detect additional simultaneously active sources at other solution points. These distributed activations may be more or less intense across microstates. To detect these distributed activations over all solution points, we first performed a qualitative visual inspection of all solution points. Then, to statistically validate whether (or not) these qualitative modulations of brain activations were significantly different between microstates, we conducted a supplementary statistical analysis. In this supplementary analysis, we contrasted the scalar values from the source estimations over every time period of one microstate (e.g., Microstate 1) for each participant (N = 20 participants) with the scalar values from the source estimations over the time period of another microstate (e.g., Microstate 2) for each participant (N = 20 participants) using a paired t-test (Bonferroni-corrected). These statistical analyses of source estimations were performed by first averaging the VEP data over such time periods of microstates in order to generate a single data point per period for each participant to increase the signal-to-noise ratio. Then the inverse solutions were estimated for each of the 3005 solution points. Only solution points with *p*≤0.05 (*t*(19)≥2.09) were considered significant. In addition, we applied a cluster threshold of >10 contiguous activated solution points.

## Results

### No Context condition

Figure 2 shows the average VEPs elicited by the presentation of the three classes of stimuli in “No Context” condition: Use grip (Ug), Transport grip (Tg), and Simple contact (Sc).

The hierarchical cluster analysis of the VEP topographies in this “No Context” condition identified five different microstates in the 500 ms post-stimulus period that explained 96.41% of variance in the collective data set. The windows of occurrence for these stable topographies corresponded to the following time intervals: Microstate 1: 0–120 ms; Microstate 2: 122–200 ms; Microstate 3: 202–230 ms; Microstate 4: 232–320 ms; Microstate 5: 322–500 ms.

The first two microstates (Figure 2 A, pale gray and dark gray bars respectively) did not significantly differ one from another as a function of the type of stimuli: Microstate 1 = 119 ms for Ug, 114 ms for Tg, and 114 ms for Sc; *F*(2,38) = 3; *p* = 0.06; Microstate 2 = 65 ms for Ug, 70 ms for Tg, and 66 ms for Sc; *F*(2,38) = 0.82, *p* = 0.45. As shown on Figure 2A, this was not the case for the next microstate, Microstate 3 (Figure 2A, blue bars). To statistically validate whether this Microstate 3 differed according to stimulus category, the values related to its duration (i.e., values that represent the frequency with which this given microstate was observed within this given time period for each experimental condition) were subjected to a repeated measure ANOVA with the within-subject factor of stimulus category (Figure 2A). This ANOVA revealed that the duration of this Microstate 3 was significantly longer in Ug = 28 ms and Tg = 26 ms (i.e. during processing of stimuli showing hand grasping) than in Sc (i.e., in the case of simple contact with the object; Sc = 18 ms; *F*(2,38) = 4.05; *p* = 0.03).

A similar ANOVA performed on the following microstate, Microstate 4 (Figure 2A, green bars), also showed a significant different duration according to the presented stimuli (*F*(2,38) = 5.3, *p* = 0.009). More precisely, Microstate 4 was significantly shorter for Ug (i.e. for the grip that is typically used to interact with the presented object) than for Tg (i.e. the grip used to move the object), and Sc. Its duration for Ug, Tg, and Sc was 59 ms, 76 ms and 84 ms, respectively. Finally, the last microstate, Microstate 5 (Figure 2A, yellow bars), did not significantly differ in function of the presented stimuli.

Figure 2B shows the segmentation maps of Microstates 3 and 4 (blue and green frames, respectively) as revealed by the topographic pattern analysis across the group-averaged VEPs of all three classes of used stimuli. Blue areas depict negative potentials, while red areas depict positive potentials. The reliability of these microstates at the group-averaged level was assessed at the individual level using a fitting procedure (Figure 2C, see Methods for further details)

Next, we estimated the active intracranial generators of every microstate identified above in the “No Context” condition (Figure 2D). To do so, we used a distributed source inverse solution (LORETA), and applied it to the topographic configurations identified in the VEP analysis. This approach allows us to define the brain areas activated in the different microstates. The active areas are shown in Figure 2D. The color scale (Figure 2 D, right part) indicates the current source density.

During Microstate 1 (pale grey frame) LORETA distributed source inverse solution revealed a bilateral activation of the occipital, posterior temporal and inferior parietal cortices with a current source density maximum located in the left middle temporal sulcus (current source density maximum: −48, −61, 6 x, y, z; Talairach coordinates).

The next microstate (Microstate 2, dark grey frame) was characterized by a current source density maximum in the caudal part of the left inferior parietal lobule (current source density maximum: −50, −58, 23 x, y, z; Talairach coordinates). Visual inspection of the other neural generators in these two microstates (Microstate 1 and Microstate 2) indicates a marked decrease of activations of the right hemisphere in Microstate 2 relative to those observed for Microstate 1 with a simultaneous accentuation of activations of the left hemisphere activity and in particular of the inferior parietal lobule. To statistically validate whether (or not) this qualitative decrease of activation in the right hemisphere in Microstate 2 was significantly different from Microstate 1, we conducted a paired t-test over the possible 3005 brain solution points (see Method section for further details). More precisely, we contrasted the scalar values from the source estimations over the 0–120 ms period (i.e., time period of Microstate 1) for each participant (N = 20 participants) with the scalar values from the source estimations over the 122–200 ms period (i.e., Microstate 2 time period) for each participant (N = 20 participants) using a paired t-test (Bonferroni-corrected) and applying a brain solution point-level significance threshold of *t*19≥2.09 (*p*≤0.05) and a cluster threshold of >10 contiguous activated brain solution points. Estimated sources in right hemisphere were significantly stronger in Microstate 1 period of time as opposed to Microstate 2 period of time (*t* = 4.84 in right inferior parietal lobule; *t* = 2.50 in right superior temporal cortex). Also, estimated sources in left inferior parietal lobule were significantly stronger in Microstate 2 period of time as opposed to Microstate 1 period of time (*t* = 6.79).

LORETA estimation of the active intracranial generators of Microstate 3 (blue frame) showed a current source density maximum located in the left inferior parietal lobule (current source density maximum: −49, −63, 17 x, y, z; Talairach coordinates). Visual inspection of the other neural generators of Microstate 3 revealed a right hemisphere parietal activation, which was not present in Microstate 2. To statistically validate whether (or not) this right brain activation was significantly different between Microstate 3 and Microstate 2, we contrasted the scalar values from the source estimations over the time period of Microstate 3 for each participant with the scalar values from the source estimations over the time period of Microstate 2 for each participant over the possible 3005 brain solution points (using a paired t-test, Bonferroni-corrected; with the following significance criteria: *t*19≥2.09 (*p*≤0.05) and cluster threshold of >10 contiguous activated brain solution points). Estimated sources confirmed a significant increase of right hemispheric activations in the inferior parietal (*t* = 3.44) and posterior temporal (*t* = 3.30) cortices in Microstate 3 period of time as compared to Microstate 2 period of time.

LORETA estimation of the active intracranial generators of Microstate 4 (green frame) also revealed a current source density maximum in the left inferior parietal lobule (current source density maximum: −48, −62, 12 x, y, z, Talairach coordinates). In addition, visual inspection of the other activations found in Microstate 4 revealed also a clear right inferior parietal and posterior temporal activation, and a bilateral activation of the frontal lobes. To statistically validate these visual observations, we contrasted the scalar values from the source estimations over the time period of Microstate 4 for each participant with the scalar values from the source estimations over the time period of Microstate 3 for each participant over the possible 3005 brain solution points (using a paired t-test, Bonferroni-corrected; with the following significance criteria: *t*19≥2.09 (*p*≤0.05), and cluster threshold of >10 contiguous activated brain solution points). Estimated sources confirmed significantly stronger right hemispheric activations in the inferior parietal (*t* = 2.42) and the superior parietal areas (*t* = 3.65) in Microstate 4 compared to Microstate 3. Also, although significantly stronger activations were present bilaterally in the frontal lobe in Microstate 4 (compared to Microstate 3), these activations did not pass our cluster threshold of >10 contiguous activated brain solution points (right frontal activations: *t* = 2.80 with 3 activated solution points; left frontal activations: *t* = 2.25 with 2 activated solution points). Finally, estimated sources in left inferior parietal lobule were significantly stronger in Microstate 3 period of time as opposed to Microstate 4 period of time (*t* = 3.04).

Finally, LORETA estimation of the active intracranial generators of Microstate 5 (yellow frame) showed a current source density maximum in the left orbito-frontal cortex (current source density maximum: −3, 38, −7 x, y, z; Talairach coordinates). A visual inspection of Microstate 5 inverse solutions revealed a decrease of all the other activations mentioned previously. To statistically validate these visual observations, we contrasted the scalar values from the source estimations over the time period of Microstate 5 for each participant with the scalar values from the source estimations over the time period of Microstate 4 for each participant over the possible 3005 brain solution points (using a paired t-test, Bonferroni-corrected; with the following significance criteria: *t*19≥2.09 (*p*≤0.05), and cluster threshold of >10 contiguous activated brain solution points). Estimated sources confirmed significantly stronger right hemispheric activations in Microstate 4 in comparison with Microstate 5, notably in the right inferior parietal (*t* = 3.44), posterior temporal (*t* = 4.20), and frontal (*t* = 3.93) areas. Also, significantly stronger left hemispheric activations were present in Microstate 4 (compared to Microstate 5), notably in the left inferior parietal (*t* = 3.04), posterior temporal (*t* = 2.51), and frontal (*t* = 2.78) lobes.

### Context condition

**Figure 3:**
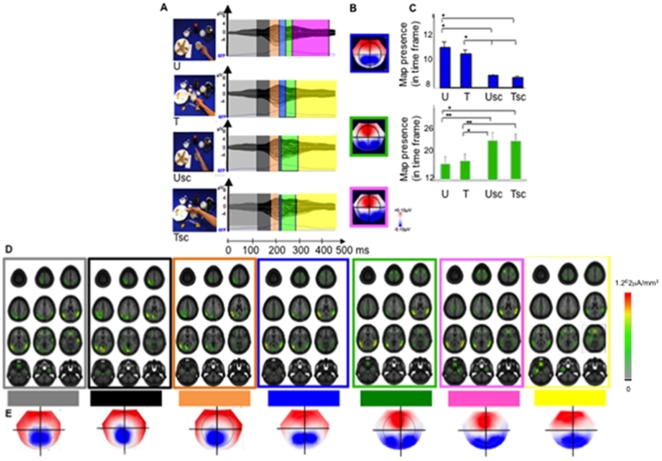
Context condition. Visual event-related potentials (VEPs) and brain microstates. **A** Group-averaged VEPs elicited by the presentation of hand grasping stimuli in use (U) and transport (T) context, and by the presentation of simple contact stimuli in the same two contexts (Usc and Tsc). Stimulus exemplars of the four classes of stimuli are shown on the left side of the A panel. The topographic cluster analysis identified six distinct microstates (colored bars) in the 500 ms following stimulus presentation. Two microstates (blue and green bars, respectively), although present following presentation of all three types of stimuli, had different duration depending upon stimulus type. The last microstate (Microstate 6, yellow bar) was identical for T, Usc and Tsc. It was markedly different for U (see text). **B**. Segmentation maps of the two microstates (Microstates 4, blue; Microstate 5, green, upper part of the column) that showed different duration according to the presented stimulus type. The lower panel of the column shows segmentation map of Microstate 6 (pink) that is specific for the case of U class of stimuli. **C**. The statistical significance of Microstate 4 and 5 was tested by means of a fitting procedure based on the spatial correlation between microstates obtained from the group-averaged VEPs and the single-subject VEP data. Blue columns: Microstate 4. Note the prolonged activation for grasping actions (U and T) with respect to those for simple contact actions (Usc and Tsc). Green columns: Microstate 5. Note the prolonged responses for Usc and Tsc than for U and T. Error bars indicate standard deviation. Asterisks indicate significant differences (*, *p*<0.05; **, *p*<0.01) between conditions for a given map observation. **D**. Localization of the intracranial brain generators as estimated with LORETA inverse solution. Twelve brain transverse sections are presented (z coordinates as in Figure 2). The localizations are framed with the same color as the corresponding microstates in A. Group-averaged sources estimations were calculated over each time interval and all conditions. **E**. Segmentation maps of the all microstates. Conventions as in B.

Figure 3A shows the average VEPs elicited by the presentation of the four classes of stimuli in the “Context” condition: Use (U), Transport (T), and Simple contact in use and transport scenes (Usc and Tsc).

The hierarchical cluster analysis of the VEP topographies in this “Context” condition identified six stable microstates in the 500 ms post-stimulus period that explained 96.54% of variance in the collective data set. While the first three microstates (0–120 ms; 122–170 ms; 172–208 ms) were identical across the four classes of stimuli (Figure 3A pale gray, dark gray, and orange bars, respectively), the next microstate, Microstate 4 (from 210–230 ms; Figure 3A blue bars), was evident only for U and T classes of stimuli. To statistically validate whether this Microstate 4 differed according to stimulus category, the values related to its duration were subjected to a repeated measure ANOVA with the within-subject factor of stimulus category (Figure 3A). This ANOVA revealed that the duration of Microstate 4 was significantly longer for the VEPs of U and T classes of stimuli (in comparison with the other classes of stimuli) over the time period from 210–230 ms (*F*(3, 57) = 4.11, *p* = 0.01). Its duration was identical for both of these classes. Microstate 4 was followed by a microstate, Microstate 5 (from 232–280 ms, Figure 3A green bars) that, although present during processing of all four classes of stimuli, had a significantly much shorter duration for U and T than for Usc and Tsc (*F*(3, 57) = 3.23, *p* = 0.03). The following microstate, Microstate 6 (from 282–450 ms, Figure 3A pink bars) was only present during the processing of use grip (U). The processing of the other three classes of stimuli was characterized by a microstate, Microstate 7 (from 282–500 ms, Figure 3A yellow bars), significantly different from Microstate 6 (*F*(3, 57) = 8.53; *p*<0.001).

The segmentation maps of Microstates 4, 5 and 6, revealed by the topographic pattern analysis for each one of the four classes of stimuli are shown in Figure 3B (blue, green and pink frames, respectively). The reliability of these microstates at the group-averaged level was assessed at the individual level using a fitting procedure (Figure 3C).

Next, we estimated the active intracranial generators of every microstate described above in the “Context” condition (Figure 3D) using the distributed source inverse solution LORETA. The estimations of group-averaged brain sources were calculated over each time period (Microstates 1–7) and class of stimuli (U, T, Usc, and Tsc). Figure 3D shows brain transverse sections displaying the local source density maxima, localized within a larger activation cerebral network observed for each microstate.

During Microstate 1 (Figure 3D, pale gray frame) LORETA distributed source inverse solution revealed a bilateral activation of the occipital, posterior temporal, and inferior parietal cortices with a left-lateralized current source density maximum (current source density maximum: −49, −73, 12 x, y, z mm Talairach coordinates). A qualitative visual inspection of these data revealed an additional activation in the anterior part of the right superior temporal sulcus (57, −44, 17 x, y, z; Talairach coordinates) in Microstate 1. Microstate 2 (Figure 3D, dark grey frame) was characterized by a left-lateralized current source density maximum (current source density maximum: −9, −84, 1 x, y, z; Talairach coordinates). A qualitative visual inspection of this Microstate 2 revealed a marked decrease of activations of the right hemisphere in comparison with those observed in Microstate 1. To statistically validate whether this qualitative decrease of activation in the right hemisphere was significantly different between Microstate 2 and Microstate 1, we conducted a paired t-test over the possible 3005 brain solution points (see Method section for further details). More precisely, we contrasted the scalar values from the source estimations over the 0–120 ms period (i.e., time period of Microstate 1) for each participant (N = 20 participants) with the scalar values from the source estimations over the 122–170 ms period (i.e., Microstate 2 time period) for each participant (N = 20 participants) using a paired t-test (Bonferroni-corrected). We applied a brain solution point-level significance threshold of *t*19≥2.09 (*p*≤0.05) and a cluster threshold of >10 contiguous activated brain solution points. As expected, estimated sources in right posterior STS were significantly stronger (*t* = 3.40) in Microstate 1 period of time as opposed to Microstate 2 period of time.

LORETA estimation of the active intracranial generators of the next microstate (i.e., Microstate 3, Figure 3D, orange frame) presented an activation pattern similar to that of the previous one (i.e., Microstate 2) with a left-lateralized current source density maximum (current source density maximum: −49, −67, 17 x, y, z; Talairach coordinates). Visual inspection of Microstate 3 (in comparison with Microstate 2) revealed a marked decrease of occipital activations and, conversely an increase of those located in the left temporal and inferior parietal cortices. To statistically validate whether (or not) these qualitative modulations of brain activation were significantly different between Microstate 3 and Microstate 2, we contrasted the scalar values from the source estimations over the time period of Microstate 3 for each participant with the scalar values from the source estimations over the time period of Microstate 2 for each participant over the possible 3005 brain solution points (using a paired t-test; Bonferroni-corrected). As previously, we applied a brain solution point-level significance threshold of *t*19≥2.09 (*p*≤0.05) and a cluster threshold of >10 contiguous activated brain solution points. Estimated sources confirmed a significant decrease of occipital activations (*t* = 2.98) and a significant increase of activations in the left temporal (*t* = 5.11) and inferior parietal (t = 4.98) cortices in Microstate 3 period of time as opposed to Microstate 2 period of time. For Microstate 4 (Figure 3D, blue frame) LORETA distributed source inverse solution showed a current source density maximum in the left inferior parietal lobules (current source density maximum: −54, −61, 11 x, y, z; Talairach coordinates). Visual inspection of the other neural generators found in Microstate 4 suggested that, unlike Microstate 3, activations, although weak, were present also in the right hemisphere (posterior temporal and inferior parietal cortices), and bilaterally in the frontal lobe. To statistically validate these visual observations, we contrasted the scalar values from the source estimations over the time period of Microstate 4 for each participant with the scalar values from the source estimations over the time period of Microstate 3 for each participant over the possible 3005 brain solution points (using a paired t-test; Bonferroni-corrected). Estimated sources confirmed significantly stronger activations in the right posterior temporal (*t* = 3.13) and inferior parietal areas (*t* = 2.46) in Microstate 4 compared to Microstate 3. Although significantly stronger activations were present bilaterally in the frontal lobe in Microstate 4 (compared to Microstate 3), these activations did not pass our cluster threshold of >10 contiguous activated brain solution points (right frontal activations: *t* = 3.26 with 6 activated solution points; left frontal activations: *t* = 2.67 with 3 activated solution points). Finally, this statistical analysis also revealed stronger activation in the left lingual gyrus in Microstate 3 compared to Microstate 4 (*t* = 2.87).

As shown on Figure 3D, Microstate 5 revealed a marked diffusion of activations to the right hemisphere (Figure 3D, green frame), though a local current source density maximum remained in the left hemisphere (current source density maximum: −48, −62, 7 x, y, z mm Talairach coordinates). A paired t-test (Bonferroni-corrected) contrasting the scalar values from the source estimations over the time period of Microstate 5 for each participant with the scalar values from the source estimations over the time period of Microstate 4 for each participant reinforced these results. Estimated sources were stronger in the right hemisphere (*t* = 2.65) in the superior temporal and inferior parietal areas in the Microstate 5 time period in comparison with the Microstate 4 time period. Stronger left-lateralized brain activations were also observed in Microstate 4 compared to Microstate 5 (*t* = 5.15 in left posterior superior temporal sulcus and lateral temporo-parieto-occipital area). As mentioned above the final microstate showed a significant different topographical pattern of activation (Microstate 6) during processing of U class of stimuli with respect to the processing of T, Usc, and Tsc classes (Microstate 7). Microstate 6 was significantly different from Microstate 7(*F*(3, 57) = 8.53; *p*<0.001). In the case of U class of stimuli (Microstate 6, Figure 3D, pink frame) LORETA distributed source inverse solution revealed a right hemispheric current source density maximum in the parieto-temporal areas (current source density maximum: 56, −43, 12 x, y, z; Talairach coordinates). In contrast, the Microstate 7 present in T, Usc and Tsc (Figure 3D, yellow frame) showed a left-lateralized current source density maximum in the orbito-frontal cortex (current source density maximum: −3, 33, −12 x, y, z mm Talairach coordinates).

## Discussion

The aim of the present experiment was to study the temporal dynamics of cortical activations during the observation of hand actions in individuals that were instructed to understand the intention of an agent interacting with objects. The technique adopted was a high-density electrical neuroimaging.

The results showed a complex but very consistent pattern of activations. In both “No Context” and “Context” conditions, the electrical neuroimaging analysis revealed *four* major steps: 1) a diffuse bilateral posterior cortical activations (Microstate 1, 0–120 ms in “No Context” and “Context” conditions); 2) a marked activation in the *left* posterior temporal and inferior parietal cortices with almost a complete *disappearance* of activations in the right hemisphere (Microstate 2, 122–200 ms in “No Context” condition; Microstates 2–3, 122–208 ms in “Context” condition); 3) a significant increase of activation of the right temporo-parietal region (Microstates 3 and 4 in the “No Context” condition; Microstates 4 and 5 in “Context” condition) in addition of the simultaneously co-active left hemispheric sources, plus a discrete bilateral frontal activation. In this step the duration of microstates differed depending on the intentional transparency of the motor acts: the observation of grasping objects for use and transport determined a more prolonged activations than touching objects and the same occurred for use grip relative to transport grip; 4) a significant global decrease of cortical activity. During this last step (with the exception of “Context” condition, use grip, where a pattern similar to that of step 3 persisted) an activation of the orbito-frontal cortex occurred. This activation could be related to an internal reward due to task accomplishment. However, because this late effect is outside the main purpose of this study it will be not considered further in the discussion.

What might be the explanation of this dynamic pattern of activations? What cognitive processes does it reflect? A precise response to this question is obviously very difficult. However, a theoretical analysis of the processes necessary for the comprehension of agent's intentions and the extant brain imaging and clinical data on the functional properties of the cortical areas active in the present study allow one to formulate some precise hypotheses.

Broadly speaking, there are two main “computational” processes that ought to take place to understand the agent's motor intention in a task as that of the present experiment: a) the recognition of the observed motor acts and its relation with the object semantics, i.e., what the agent is doing (e.g. “the cup is grasped by its handle with a precision grip”); b) the comprehension of *why* the cup is grasped in that specific way (e.g.“ the agent grasping the cup to drink”). These two processes, although strictly related, occur, at least in part, serially. If the motor act is not analyzed, the intention behind it cannot be understood.

Let us now examine how the temporal dynamics of the cortical activations found in the presented study fits with recognition processes.

### Left hemisphere activity and its role in motor act understanding

The first striking event in the temporal dynamics of activations leading to intention understanding is the occurrence, after an initial *bilateral* activation (step 1), of activation of the *left hemisphere* (step 2). How can this left dominance be explained?

Left hemisphere is the hemisphere specifically involved in action organization. This notion goes back to Liepmann (1900), who first showed that damage to the left inferior parietal lobule and/or the left premotor cortex produces higher-order motor deficits known as apraxias [52], the symptomatology of which (e.g. ideational apraxia, ideomotor apraxia, mielokynetic apraxia, buccofacial apraxia) varies according to the sector of the parieto-frontal network that is damaged [53], [54].

Some patients with ideomotor apraxia also present deficits in action recognition [55], [56]. In this regard particularly interesting is a recent study by Pazzaglia et al. (2008). These authors examined the capacity of patients with limb or buccofacial ideomotor apraxia to recognize hand and mouth action-related sound [57]. They found that patients with limb apraxia were impaired in recognizing hand action-related sounds, while those with bucco-facial apraxia has difficulty in recognized the sound of mouth actions. Lesion mapping revealed that crucial for recognizing the sound of limb movements was the left parieto-frontal cortex, while the left inferior frontal gyrus and the adjacent insular cortex were causatively associated with recognition of bucco-facial-related action sounds. This double dissociation indicates that a left-lateralized network is actively involved not only in execution but also in comprehension of limb and mouth actions (parieto-frontal mirror network).

Left hemisphere dominance in action observation is in accord with a large number of brain imaging studies showing prevalence of this hemisphere during the observation of object-directed motor acts [58], [59], [60], [61], [62]. In line with this is also a study carried out on a split-brain patient. By using transcranial magnetic stimulation (TMS) of motor cortex, Fecteau et al. (2005) found that, during action observation, the tested split-brain patient showed an enhanced excitability of the left hemisphere, while no enhancement was observed following stimulation of the right one [63].

It is worth noting that bilateral activation of the parieto-frontal mirror circuit during the observation and imitation of finger movements was reported in an fMRI study by Aziz-Zadeh et al. [64], [65]. It is likely, however, that these findings are due to the type of motor behavior investigated. There is evidence that in humans there are two different motor networks endowed with the mirror mechanism [66], [67]. One encodes the observed movements independently of the motor act of which they are part [68], [69], [70], [71], the other encodes the goal of the observed motor acts independently of how this goal is achieved [72], [73], [74]. It is plausible that the mirror networks encoding movements and motor acts are not equally lateralized. While, the former is likely bilateral, the latter is localized to the left hemisphere.

In conclusion, these data indicate that the activation of the left inferior parietal lobule is likely due to the encoding the observed motor act on motor “engrams” present in this cortical region. In virtue of the mirror mechanism, this encoding allows the individual to recognize *what* the agent is doing. As for the concomitant left temporal lobe activation, it is very likely that it is due to the processing of the semantics of objects acted upon. To this regard there is evidence from fMRI experiments that the processing of the semantics of inanimate objects that can be manipulated (tools in particular) is influenced by their pragmatic properties and, as a consequence, is localized in the *left* temporal lobe [75].

### From left to right hemisphere

The next step in intention understanding process (step 3) is the most complex and intriguing. During this step the activation of the temporo-parietal region of the left hemisphere continues, but is accompanied by a progressively more and more intense activation of the right hemisphere. This step consists of two Microstates, the first (Microstate 3 “No Context” and Microstate 4 “Context” conditions) characterized by an initial rather weak right hemisphere activation, the left hemisphere being still prevalent; the second (Microstate 4 “No Context” and Microstate 5 “Context” conditions) during which the right hemisphere activation becomes full fledged.

It is likely that step 3 reflects cortical activations related to *intention* understanding. There are two sets of arguments in favor of this proposal. The first is based on the activation pattern observed in the present study; the second derives from brain imaging data (see below) in which attempts have been done to localize the mechanisms responsible for intention understanding.

In the “No Context” condition, the observation of the two motor acts having a transparent goal (use and transport grip) was characterized by a more prolonged topographical pattern (Microstate 3; *p* = 0.03; see Figure 2C) with a left-lateralized current source density maximum in the inferior parietal lobule (−49, −63, 17 x, y, z; Talairach coordinates; Figure 2D) than the observation of the intentionally opaque simple contact. Conversely, the observation of a simple touch produced a more prolonged activation in the next brain state (Microstate 4) than the observation of use grip, and, the observation of the touch grip a longer activation relative to transport grip (*p* = 0.009; see Figure 2C). This specific brain sate (Microstate 4) was characterized by a left-lateralized current source density maximum in the inferior parietal lobule and also with significantly stronger right hemispheric activations in the inferior parietal and superior parietal areas than in Microstate 3 (see Figure 2D). Although we cannot exclude that different conditions are explained by different brain generators, the most plausible interpretation of the difference in the duration of Microstate 3 is due to the amount of motor information contained in grasping relative to simple contact. During grasping observation, the processing of motor information leading to goal understanding requires more time because of the complexity of the observed grip and its relation with the object. This time-consuming operation does not occur in the case of simple contact, because the goal understanding here does not require a detailed analysis of the motor aspects of the hand-object interaction.

This proposal also accounts for the difference in time between different types of stimuli in the next microstate (Microstate 4). It is plausible that more detailed is the description of an observed motor act, less time is required to understand the motor intention behind it. The brief right hemisphere activation during the processing of transparent motor act could reflect this fact. This hypothesis is corroborated by a comparison of the two grip conditions: the shorter time for processing use grip relative to transport grip should reflect the congruence between the semantics of the observed object and its use, a congruence lacking in the case of an unspecific motor act as transport grip. In contrast, when, as in the case of simple contact, the motor act is poorly related to the object, the time for trying to understand the agent's intention become longer, requiring a more sophisticated analysis of the visual scene and possibly (see below) the involvement of inferential processing.

The results obtained in the “Context” condition support this interpretation of the two microstates. Also in the “Context” condition there was a longer duration of the Microstate 4 (the first state showing difference in activation between different classes of stimuli; *p* = 0.01; see Figure 3A–C) and a briefer duration of the Microstate 5 for grasping motor acts relative to simple contact actions (*p* = 0.03; see Figure 3C). The main difference with the “No Context” condition was that the use and transport grasping did not differ in time in either microstate. The most likely reason for this was that, unlike in the “No Context” condition, in the “Context” condition the relevant cue for understanding the agent's intention was not the hand-grip but the specific context in which the motor act was performed, and, as one can see from Figure 1, the two contexts did not differ in their complexity as well in their significance for intention understanding.

Finally, during the observation of use grip in the “Context” condition there was a prolonged late activation in the right hemisphere. A possible explanation of this finding is that when the action processing is based on both the use grip and use context, there is a further amplification of the activity in the right hemisphere which might reflect the elaboration by the observer of possible reasons behind agent's intentions.

Although the results of the present study cannot exclude some role of the left hemisphere in intention understanding, they clearly indicate that the right hemisphere plays an important role in this function. This conclusion has been recently supported by a study, prompted by the results of the present experiment, carried out on a split-brain patient [76]. This patient was tested in two conditions: “means inference” task and “intention inference” task. In both tasks stimuli similar to those of the present study were used, but in one the patient was required to guess if the means of the observed act was correct, in the other if the intention was correct. The responses were done either with the right hand (left hemisphere) or the left hand (right hemisphere). Results from this split-brain patient showed a left hemisphere dominance for understanding “what” is done and a right hemisphere dominance for understanding “why” an action is carried out.

These findings are in line with previous fMRI data by Iacoboni et al. (2005) and Hamilton and Grafton (2008). The first study showed activation of the *right* frontal node of the mirror network in volunteers required to recognize the intention of an agent on the basis of the context in which the action was performed [20], the other, using the repetition suppression paradigm, demonstrated that both parietal and frontal nodes of the *right* hemisphere mirror network are involved in motor intention understanding [21].

The inverse solution of source localization on which our study is based allows us only an approximate anatomical cortical localization of the activations. Thus, we cannot assert whether the right temporo-parietal activations observed in our study during intention understanding includes only centers endowed with mirror mechanism (plus the temporal areas involved in object description) or also other temporal areas devoid of this mechanism. In this respect is important to note that there is evidence that the observation of other's actions, in particular when they are unusual or non-stereotypical, might determine, in addition to the activation of the mirror network, the activation of the posterior part of the right superior temporal sulcus (pSTS) [13], [14], [16]. The activation of the right STS in “unusual” conditions could be due to a division of labor between the two hemispheres according to the type of visual stimuli that is analyzed. While typical effector-object interactions are processed by the left hemisphere, the processing of uncommon interactions lacking specific motor engrams in the parieto-frontal mirror networks, is function of the right pSTS.

### Conclusion

The notion that the right hemisphere is involved in motor intention understanding, regardless of whether through the mirror mechanism or higher- order visual mechanisms, may have also interesting implications for the comprehension of the relation between the observation of the behavior of others and the mentalizing processes by means of which the observer attribute to others specific mental states. Over the last few years, fMRI studies suggested that specific parts of the right hemisphere and in particular the right parieto-temporal junction (TPJ) are critically involved in belief [11], [77] and agency [78] attribution. Keeping this in mind, the prevalence of right hemisphere in motor intention understanding, especially in the case of actions whose intention is not transparent and might require reasoning for decipher it, could be the bridge linking motor act recognition with higher-order mentalizing processes such as belief and agency attribution.

## Supporting Information

File S1 Supporting Information.
(0.03 MB DOC)

Figure S1 Brain microstates and LORETA distributed linear inverse solution estimation for data time-locked to the first picture of context and no context conditions.
(0.73 MB TIF)
