# Supplementary material for: Understanding Actions of Others: The Electrodynamics of the Left and Right Hemispheres. A High-Density EEG Neuroimaging Study
Source: PLoS One. 2010 Aug 13;5(8):e12160. doi: 10.1371/journal.pone.0012160 (PMC2921336; doi:10.1371/journal.pone.0012160)
Supplement: File S1 — Supporting Information. (0.03 MB DOC) [file pone.0012160.s001.doc]

**Supporting Material (SM).**

**Supporting Results**

Electrical neuroimaging results for data time-locked to the first picture (T0) of “Context” and “No Context” conditions. Archetypal VEP components (e.g., P1 and N1) were readily observed for each condition (i.e., object presented without context; object presented with a use contextual background and object presented with a transport contextual background; Figure S1). A P1 first peaked at approximately 130 ms on average, and then an N1 peaked at approximately 180ms on average (N1). N1 and P1 were maximal at temporo-occipital sites. As shown on Figure S1, the 80-140 ms scalp topography is characterized by a positive activity in the posterior region. Then, 142-210 ms scalp topography is characterized by a negative activity in the posterior region. The topographical pattern analysis identified: 0-78 ms, 80-140 ms, 142-210 ms, 212-270 ms, 272-400 ms, and 402-500 ms as stable spatial configurations (i.e., Figure S1). While most of the microstates were observed in the group-averaged VEPs of all conditions, two of them were observed less often in one condition (top row) from 212 to 400 ms, as compared to the two other conditions. First, Microstate 4 (in pink frame in Figure S1*AB*) was present from 212-240 ms in the “No Context” condition, although it was observed 30 ms longer in the “Context” condition (from 212 ms to 270 ms; *F*(2,38) = 32.503; *p* < 0.001). Then, Microstate 5 (in blue frame) started earlier (i.e., at 242 ms) in the “No Context” condition than in the two “Context” conditions (at 272 ms; *F*(2,20) = 38; *p* < 0.001). Microstate 5 lasted a shorter amount of time (i.e., 64 ms) in the “No Context” condition (top row) than within in the “Context” condition (i.e., 110 ms for object presented with a use contextual background, middle row; 104 ms for object presented with a transport contextual background, bottom row). As expected, no difference was observed among the two “Context” conditions (*F*(1,19) = 3.55; *p* = 0.08).

The maximum amplitude of the GFP was larger in the “Context” condition (U = 0.72; T = 0.70) than for the “No Context” condition (0.47) between 272 and 400 ms after stimulus onset (*F*(2,38) = 23.25; *p* < 0.001). No other differential GFP amplitude was observed earlier between conditions (e.g., for 212-270 ms; *F*(2,38) = 1.37; *p* = 0.27). The 272-400 ms effect was certainly due to the large physical differences between experimental conditions underlying the differential recruitment for scene recognition. Our assumption is reinforced by the absence of amplitude differences between the two “Context” conditions (*F*(1,19) = 3.7; *p* = 0.07).

We applied the LORETA distributed linear inverse solution estimation to visualize the brain sources underlying the different microstates (Figure S1). Figure S1 displays brain transverse sections showing current local source density maxima, localized within a larger cerebral network that was observed for each map. First, this source estimation revealed bilateral cuneus and lingual/calcarine sources (local current source density maximum at the level of Brodmann’s area 18, lingual/calcarine cortex; 3, -82, 1; x, y, z mm Talairach coordinates) for Map 1 (in black frame). Then a right lateral occipito-temporal source (local current source density maximum: 52, -59, 1; x, y, z mm Talairach coordinates) was observed for Map 2 (in white frame), followed in time by bilateral temporal sources (Map 3 in green frame; local current source density maximum: right superior temporal gyrus**,** Brodmann’s area 39: 52, -54, 11; x, y, z mm Talairach coordinates). Map 4 (in pink frame) was characterized by a bilateral (mostly right-sided) activity in the superior temporal sulcus (right superior temporal sulcus**;** local current source density maximum: 53, -55, 17; x, y, z mm Talairach coordinates), while Map 5 (in blue frame) was characterized by a bilateral (mostly left-sided) temporal and lateral occipital activity (local current source density maximum: left middle temporal gyrus: -49, -62, 12; x, y, z mm Talairach coordinates). Finally, the source estimation revealed a bilateral lateral temporo-occipital activity (Map 6 in yellow frame; local current source density maximum: left lateral temporo-occipital cortex, Brodmann’s area 37; -48, -61, 1; x, y, z mm Talairach coordinates). Taken together, the present results are in keeping with the electrophysiological literature on scenes and object perception.

**Supporting Figure Legend**

**Figure S1.** **EEG neuroimaging for** **data time-locked to T0.** (A) Samples of first picture stimuli and brain microstates elicited by their visual presentation. (B) Segmentation maps are plotted with the nasion upward and right ear on the right side (scale indicated). Blue areas depict negative potentials and red areas depict positive potentials. (C) The figure displays brain transverse brain sections showing current local source density maxima, localized within a larger cerebral network that was observed for each segmentation map.
