# Supplementary figures and images for: Understanding Actions of Others: The Electrodynamics of the Left and Right Hemispheres. A High-Density EEG Neuroimaging Study
Source: PLoS One. 2010 Aug 13;5(8):e12160. doi: 10.1371/journal.pone.0012160 (PMC2921336; doi:10.1371/journal.pone.0012160)

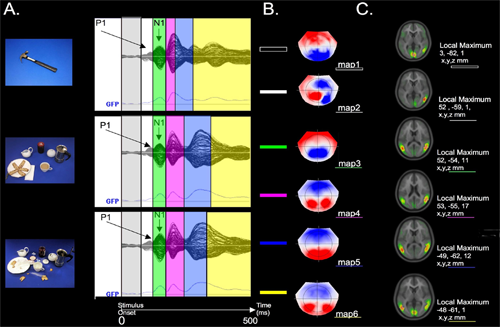

Supplement: Figure S1 — Brain microstates and LORETA distributed linear inverse solution estimation for data time-locked to the first picture of context and no context conditions. (0.73 MB TIF) [file pone.0012160.s002.tif]
